# Is Ceftriaxone-Induced Biliary Pseudolithiasis Influenced by UDP-Glucuronosyltransferase 1A1 Gene Polymorphisms?

**DOI:** 10.1155/2011/730250

**Published:** 2011-10-26

**Authors:** Andrew Fretzayas, Olga Liapi, Anna Papadopoulou, Polyxeni Nicolaidou, Alexandra Stamoulakatou

**Affiliations:** ^1^Third Department of Pediatrics, “Attikon” General University Hospital, 1 Rimini Street, Haidari, 124 62 Athens, Greece; ^2^Laboratory of Hematology, “Aghia Sophia” Children's Hospital, Thivon and Papadiamantopoulou Street, Goudi, 115 27 Athens, Greece

## Abstract

Ceftriaxone (cfx), a third-generation cephalosporin antibiotic, leads to transient cholelithiasis in some children, also known as pseudolithiasis. However, the underlying pathogenetic mechanism of this adverse effect has not yet been elucidated. We describe 3 children with ceftriaxone-induced pseudolithiasis, who were also carriers of the A(TA)_7_TAA polymorphism of the *UGT1A1* gene, implying that a cause and effect relation may exist.

## 1. Introduction

Cholelithiasis in childhood is usually considered a secondary effect of various predisposing factors such as hemolytic anemia, hepatobiliary diseases, total parenteral nutrition, sepsis, bowel resection, or a side effect of certain medications [1, 2]. Among the latter, ceftriaxone (cfx), originally described by Schaad et al. [3], has been recognized as a lithogenic substance in a proportion of treated patients. In these cases, fine precipitations or sludge are formed temporarily, resembling gallstones. They resolve shortly after stopping cfx, and various terms, such as “pseudolithiasis” or “reversible lithiasis”, have been used [4].

As the pathogenesis of cfx-induced pseudolithiasis is currently unclear, and main predisposing factors, that is, the drug and the infection are common in all patients, the answer might be hidden in the patient's genomic data. We observed 3 children with cfx-induced pseudolithiasis who were also carriers of the A(TA)_7_TAA polymorphism of the *UGT1A1* gene. *UGT1A1* encodes UDP-glucuronosyltransferase (UDPG), an enzyme engaged in the glucuronidation pathway that transforms small lipophilic molecules, (i.e., steroids, bilirubin, hormones, and drugs) into water-soluble, excretable metabolites.

## 2. Case Presentation

Our 3 patients, consisting of one girl and two boys, aged 5,5 months, 18 months, and 4 years old, respectively, were treated for urinary tract infection and received cfx (100 mg/kg/day) IV for 10–14 days. One patient also received cefotaxime prior to cfx treatment. None had a history of predisposing factors, a family history of gallstones, or was treated with drugs associated with gallbladder lithiasis, such as octreotide or furosemide. 

**Figure 1:**
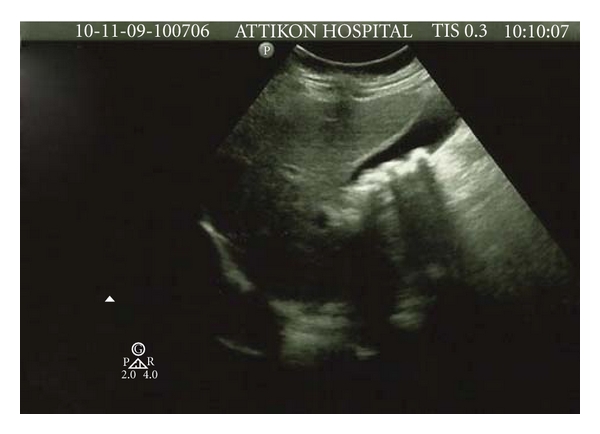
Sonogram in one of our patients showing multiple echogenic material with acoustic shadowing.

All three patients developed gallbladder pseudolithiasis with typical sonographic appearance: intense, mobile, echogenic material with acoustic shadow (Figure 1). They were repeatedly screened with gallbladder ultrasound (US) while on treatment, and follow-up examination was scheduled weekly until resolution of pseudolithiasis was seen. Extensive laboratory investigation revealed normal blood cell count without overt hemolysis, liver and renal function tests within normal limits, as well as serum lipids and electrolytes. Extensive details of the patients' data can be found in Table 1.

Parents gave informed consent prior to the inclusion of their children in the study. Genomic DNA for TA insertion on the TATAA box of the *UGT1A1* gene was analyzed, as previously described [5]. Molecular analysis of the TATAA-box-like sequence of the *UGT1A1 *promoter revealed that two of the patients were heterozygous (TA_7_/TA_6_) and one homozygous (TA_7_/TA_7_) for the polymorphism in the TATAA box in the promoter region of the *UGT1A1* gene. The study was approved by the appropriate ethics committee and has therefore been performed in accordance with the ethical standards.

## 3. Discussion

Although several prospective studies have evaluated the incidence of transient gallbladder pseudolithiasis in patients treated with cfx, the underlying pathogenetic mechanism has not been elucidated as yet [3, 4, 6]. Of interest, our children with cfx-induced pseudolithiasis share a common denominator that is hetero-/homozygosity for the A(TA)_7_TAA polymorphism of the *UGT1A1* gene, implying that a cause and effect relation may exist. In fact, the UDPG encoded by the *UGT1A1 *gene plays an important role in the handling of anionic substances in general, and in bilirubin elimination in particular. Furthermore, UDPG acts on glucuronidation and formation of bile salts [7].

It has long been recognised that the clinical sequence of reduced *UGT1A1* expression and activity of the relevant enzyme is Gilbert syndrome with diverse percentages in different populations. Indeed, a previous study has shown that DNA polymorphisms of extra TA nucleotides in the repetitive TATAA box of the promoter region of *UGT1A1* gene in Greek population are prevalent [8]. 

Numerous studies have tried to clarify the underlying pathogenesis of cfx-induced pseudolithiasis [6, 9]. Beginning with the chemical composition of fine lithogenic precipitations, it should be stated that they are mainly consisted of cholesterol monohydrate and calcium bilirubinate. It is also known that cholesterol precipitates when the cholesterol-solubilizing power of bile acid mixed micelles and phospholipid vesicles is overwhelmed. In addition, bilirubin is excreted as a soluble diglucuronide and deconjugation, either by nonenzymatic hydrolysis or by P-glucuronidase, results in free bilirubin. When the solubility product of calcium and unconjugated bilirubin is exceeded, calcium bilirubinate precipitates [10]. 

Nevertheless, how cfx has been involved in these mechanisms remains an unanswered question. This drug is a small, anionic, negatively charged organic molecule, mainly eliminated in the urine, and to a lesser extent (40%) excreted into bile [11]. Physicochemically, it is expected that when the solubility product of calcium and cfx is exceeded, precipitation should follow. Precipitation of a drug or a xenobiotic in the biliary system is rarely seen; however, as cfx is an organic anion, it behaves like other calcium-sensitive anions in bile (carbonate, bilirubinate, phosphate, palmitate) that are implicated in the pathogenesis of gallstones [10]. It has been found that cfx and bile acids share a common mechanism for hepatic transport and biliary excretion. It has also been proved that high doses of cfx are more likely to cause pseudolithiasis [9]. However, only a part of patients treated with the maximum dose develop pseudolithiasis, while pseudolithiasis has also been observed in lower doses [10]. On the other hand, considering that the drug's handling in all patients follows a defined process, and what is more, the infectious agent itself does not seem to deter the physicochemical consistency and properties of the drug, but only affects the volume distribution and tissue penetration, we assume that the factor determining the biliary sludge formation is not the infection. As a consequence, the fact that only a number of cfx-treated patients is subject to gallbladder precipitations indicates that predisposed individuals do exist. In addition, as there are no statistically important differences in the characteristics and phenotypes of the affected group (age, gender, etc.), the differentiating factor in this clinical expression must be hidden in patients' genes. For this reason, we have already begun a prospective study in which we follow-up children hospitalised in our department and treated with cfx, studying with molecular analysis the genomic regions with high incidence of the *UGT1A1* gene polymorphisms.

In conclusion, we presented three patients with cfx-induced pseudolithiasis and provided suggestions for this occurrence among which the reduced function of UDPG due to *UGT1A1* gene polymorphisms seems to be the most compatible. 

## Figures and Tables

**Table 1:** Data about the clinical and family history and lab results (inflammation markers and liver function) of the patients.

	Patient #1	Patient #2	Patient #3
Clinical complications			
Pain	Not referred	Intense abdominal pain	Not referred
Others	Not referred	Not referred	Not referred
History of prolonged jaundice after birth	Not referred	Not referred	Not referred
Family history of gallstones and Gilbert (Meulengracht disease)	Not referred	Not referred	Not referred
Other genetic variations	Not referred	G6PD deficiency	Not referred

Lab results			
Inflammation markers			
WBC (/*μ*L)	16.170	18.240	14.690
NEUT-LYMPH-MONO (%)	94-4-2	65-29-6	48-43-3
CRP (mg/L)	15.5	19.9	45.90

Liver function			
SGOT (U/L)	28	30	24
SGPT (U/L)	12	13	17
ALP	124	135	172
*γ*GT (U/L)	9	14	10
TBIL (mg/dL)	0.41	0.3	0.2
DBIL (mg/dL)	0.13	0.11	0.1

WBC: white blood cell count, NEUT: neutrophils, LYMPH: lymphocytes, MONO: monocytes, CRP: C- reactive protein, SGOT: serum glutamic-oxaloacetic transaminase, SGPT: serum glutamic-pyruvic transaminase, ALP: alkaline phosphatase, *γ*GT: gamma-glutamyl transpeptidase, TBIL: total bilirubin, DBIL: deconjucated bilirubin.

## Abbreviations

Cfx: Ceftriaxone
*UGT1A1*: UDP-glucuronosyltransferase 1 family polypeptide A1
UDPG: UDP-glucuronosyltransferase
US: Ultrasound.
